# Effect of Fenofibrate on Markers of Gut Barrier Function in Dogs With Naturally Occurring Diabetes Mellitus

**DOI:** 10.1111/jvim.70125

**Published:** 2025-05-19

**Authors:** Allison L. O'Kell, Jocelyn Mott, Lauren Porter, Chiquitha D. Crews, Yu‐An Wu, Rosemary Walzem, Joerg M. Steiner, Chen Gilor

**Affiliations:** ^1^ Department of Small Animal Clinical Sciences University of Florida Gainesville Florida USA; ^2^ Gastrointestinal Laboratory, Department of Small Animal Clinical Sciences, College of Veterinary Medicine and Biomedical Sciences Texas A&M University College Station Texas USA; ^3^ Department of Poultry Sciences, Graduate Faculty of Nutrition Texas A&M University College Station Texas USA

**Keywords:** fibroblast growth factor‐21, lipopolysaccharide, pancreatic lipase immunoreactivity, pancreatitis, peroxisome proliferator‐activated receptor

## Abstract

**Background:**

Fenofibrate improves gut barrier function and reduces serum lipids in purpose‐bred dogs with induced diabetes mellitus (DM), but its effects in dogs with naturally occurring DM are unknown.

**Objectives:**

Determine the effects of fenofibrate on markers of systemic and pancreatic inflammation, markers of gut barrier function, lipoprotein profiles, and glycemic control in dogs with naturally occurring DM.

**Animals:**

Sixteen client‐owned dogs with naturally occurring, uncomplicated DM.

**Methods:**

Longitudinal cohort study. Dogs were treated with fenofibrate (Tricor, 6–10 mg/kg, P.O., once daily) for 21 days. Interstitial glucose, serum cytokines, lipopolysaccharide (LPS), pancreatic lipase, and lipid profiles were compared between baseline and day 21 using paired *t*‐tests and Wilcoxon signed‐rank tests.

**Results:**

Fenofibrate had no effect on glycemic control, serum cytokines, or serum pancreatic lipase. Compared to baseline, the concentrations of serum LPS decreased at day 21 by (mean ± SD) 15 ± 24% (95% CI 2–28%, *p* = 0.03), serum triglycerides decreased by 36 ± 39% (95% CI 15–56%, *p* = 0.002), and serum cholesterol decreased by 20 ± 14% (95% CI 12–28%, *p* < 0.0001).

**Conclusions and Clinical Importance:**

Fenofibrate treatment was not associated with a decrease in markers of systemic or pancreatic inflammation. In diabetic dogs, short‐term fenofibrate treatment appears to be safe, and the improvement in gut barrier function and lipid profiles might lead to long‐term benefits, such as reduction in pancreatitis risk and frequency of signs of gastrointestinal disease.

**Abbreviations**
AUC: area‐under‐the‐curve
cPLI: canine pancreatic lipase immunoreactivity
DKA: diabetic ketoacidosis
DM: diabetes mellitus
EPI: exocrine pancreatic insufficiency
FGF‐21: fibroblast growth factor‐1
FGMS: flash glucose monitoring system
HDL: high‐density lipoprotein
LDL: low‐density lipoprotein
LPS: lipopolysaccharide
PPAR: peroxisome proliferator‐activated receptor
RI: reference interval
Spec cPL: specific canine pancreatic lipase
TRL: triglyceride‐rich lipoprotein

## 1 Introduction

The etiology of diabetes mellitus (DM) in dogs is likely multifactorial, with pancreatitis being a possible contributor to pathogenesis in some cases and/or a common co‐morbid condition [1, 2, 3]. Hyperlipidemia is a known sequela of DM in dogs and is suggested as a risk factor for pancreatitis [4, 5]. Intestinal and systemic inflammation are documented in diabetic dogs [6], and hyperglycemia is linked to gut barrier dysfunction and systemic inflammation in dogs [7, 8]. Along with insulin, therapies directed at improving gut barrier function, decreasing pancreatic and systemic inflammation, and treating hyperlipidemia have the potential to reduce complications and co‐morbid conditions in dogs with DM. The fibric acid derivative fenofibrate is a compound that holds promise in this area and is in clinical use for the treatment of hypertriglyceridemia in dogs [9].

Fibrates activate the peroxisome proliferator‐activated receptor (PPAR)α which, among other mechanisms, restores the integrity of the intestinal barrier during chronic gut inflammation in a primate model of intestinal dysfunction [10]. Dogs with streptozotocin‐induced DM have altered gut epithelium characterized by lower expression of tight‐junction proteins and a marked infiltration of the epithelium with lymphocytes. With fenofibrate treatment, these alterations were reversed and there was a 30% reduction in serum TNFα and IL‐8 [7].

Pancreatitis is diagnosed in about 40% of dogs with either uncomplicated DM or with diabetic ketoacidosis [11, 12]. Fibroblast growth factor‐21 (FGF‐21) is a hormone predominantly expressed in hepatocytes in response to ketosis or when food is witheld [13], but is also highly expressed in the exocrine pancreas, where it acts directly on acinar cells to stimulate digestive enzyme secretion and maintain pancreatic proteostasis [14]. In mice and people with pancreatitis, FGF‐21 is down‐regulated, and its restoration can reverse and prevent the disease in mouse models [15]. Importantly, FGF‐21 is significantly upregulated by fenofibrate, downstream of PPARα [13, 16, 17]. Therefore, fenofibrate has the potential to have multifactorial important benefits in naturally occurring DM in dogs.

The purpose of this study was to determine the effects of fenofibrate on markers of systemic inflammation, pancreatic inflammation, gut barrier function, lipoprotein profiles, and glycemic control in dogs with naturally occurring DM that are treated with insulin. We hypothesized that fenofibrate treatment would reduce serum lipopolysaccharide (LPS) and systemic inflammation and decrease serum canine pancreatic lipase immunoreactivity (cPLI). A secondary hypothesis was that fenofibrate would selectively reduce serum triacylglyceride concentrations and improve lipoprotein profiles by greatly decreasing absolute and relative amounts of triglyceride‐rich lipoprotein (TRL) and its metabolic end product, low‐density lipoprotein (LDL), while shifting plasma cholesterol carriage from TRL and LDL to high‐density lipoprotein (HDL). Plasma HDL was expected to increase in total amounts and also greatly increase in its relative amounts. Considering previous evidence, lipoprotein changes were not expected to affect short‐term glycemic control [7].

## 2 Materials and Methods

Dogs with uncomplicated DM were prospectively enrolled in the study between February 2021 and February 2023 if they met the following inclusion criteria: (1) neutered/spayed with a body weight of 5.0–45.0 kg, (2) aged 3–13 years, (3) diagnosed with DM based on standard criteria [18], (4) treated with insulin (any type or dose) for at least 2 months, (5) good to moderate diabetic control defined by stable body weight and minimal polyuria and polydipsia, and (6) clients willing and able to use the flash glucose monitoring system (FGMS). Dogs were excluded if they had a diagnosis (or strong suspicion) of concurrent endocrine disease (e.g., hypercortisolism, hypothyroidism, other), a history of diabetic ketoacidosis in the past 60 days, being treated with glucocorticoid medications, or if other significant concurrent disease, as assessed by the investigators, was present. The study protocol was approved by the Institutional Animal Care and Use Committee (IACUC #202011118) and the Veterinary Hospital Research Review Committee, and informed consent was obtained for all dogs prior to enrollment.

To obtain baseline data related to glycemic control, a FGMS sensor (Abbott FreeStyle Libre 2) was placed 7 days before the baseline visit. After application of 3–4 drops of tissue glue to the sensor's adhesive side, the sensor was applied to the dog's back (thoracolumbar area, an inch off the midline). If during this 7‐day period a dog had significant hypoglycemia or persistent severe hyperglycemia that necessitated an insulin dosing change for patient safety, the baseline visit was delayed until glycemic control and insulin dose were stabilized. At the baseline visit (day 0) all dogs had a complete physical examination, including body weight, complete blood count, biochemistry profile, and serum banking for measurement of study analytes. Dogs meeting the inclusion criteria were prescribed fenofibrate (FNF, Tricor, 6–10 mg/kg, PO, given once daily, with food in the evening). Owners were provided a daily dosing diary to monitor compliance as well as to note any health concerns, and were instructed to scan the FGMS sensor at least every 8 h and upload to the LibreView website to be monitored by the investigators. The FGMS sensor was replaced on day 14 by the owner, referring veterinarian, or by the study investigators. Dogs returned on day 21 for the final study visit that included a complete physical examination, including body weight, complete blood count, and a biochemistry profile. Blood was also collected and serum separated and stored at −80^o^C for measurement of study analytes. Food was withheld for 12 h prior to blood collection at the baseline and Day 21 visits.

Insulin therapy was not changed throughout the study period unless a dose decrease was required (if hypoglycemia was detected) as recommended by the attending clinician. No changes in diet or additional medications were allowed. Dogs were withdrawn from the study if they developed any major illness that required hospitalization or that caused the dog to be unable to take oral medications (e.g., anorexia, vomiting) for more than 2 days.

At baseline and day 21 visits, blood was collected and serum separated routinely and stored at −80^o^C for measurement of serum cytokines (i.e., IL‐2, IL‐6, IL‐8, and TNFα), serum LPS, serum cPLI concentration, and lipoprotein profiles. Following study completion, an aliquot of all serum samples was shipped to the Gastrointestinal Laboratory at Texas A&M University. Serum cPLI concentrations were measured using a commercially available validated assay, Spec cPL [19]. The reference interval (RI) for cPLI is 0–200 μg/L, with > 400 μg/L being consistent with pancreatitis. Lipoprotein profiles were measured using a bismuth sodium ethylenediaminetetraacetic acid density gradient ultracentrifugation method previously validated for use in dogs [20]. Briefly, density profiles of circulating lipoproteins were determined by ultracentrifugation of fluorescently labeled plasma, as detailed elsewhere [20] and analyzed as the absolute area‐under‐the‐curve (AUC) where the image area was measured in pixels (i.e., number of pixels within a density interval). A total of 11 nominal lipoprotein subclasses were identified by their density intervals, including TRL (density [*d*] < 1.019 g/mL), LDL1 (*d* = 1.019–1.023 g/mL), LDL2 (*d* = 1.023–1.029 g/mL), LDL3 (*d* = 1.029–1.039 g/mL), LDL4 (*d* = 1.039–1.050 g/mL), LDL5 (*d* = 1.050–1.063 g/mL), HDL2b (*d* = 1.063–1.091 g/mL), HDL2a (*d* = 1.091–1.110 g/mL), HDL3a (*d* = 1.110–1.133 g/mL), HDL3b (*d* = 1.133–1.156 g/mL), and HDL3c (*d* = 1.156–1.179 g/mL). Appropriate values were summed to give the total pixels (i.e., AUC) of TRL, LDL, and HDL, respectively. Fractional distribution of total lipoprotein AUC was calculated by division of lipoprotein subclass AUC by total lipoprotein AUC multiplied by 100%. Serum cytokines were measured using a commercial multiplex electrochemiluminescence immunoassay (Meso Scale Diagnostics) as previously described [21]. LPS was quantified in samples taken at baseline and on day 21 using the Pierce Chromogenic Endotoxin Quant Kit by ThermoFisher Scientific [8]. Serum samples stored at −80^o^C were thawed at room temperature and centrifuged at 4000 rpm for 7 min. Samples were then diluted 1:50 using the endotoxin‐free water provided in the kit and heat shocked at 70^o^C for 15 min. The assay was then performed according to the manufacturer's instructions, all samples were analyzed in triplicates, and the standard curve was created using the low standards protocol.

### 2.1 Statistical Analysis

To determine sample size, a power calculation was performed. Based on data in laboratory beagles with induced DM, we expected a mean ± SD reduction of 36% ± 24% in TNFα (and similar in IL‐8) from day 0 to day 21. In that study, this magnitude of effect yielded a statistically significant result (*p* = 0.03) with a sample size of 7 diabetic dogs. Here, to account for treating a more heterogeneous group of dogs, which would likely yield results with greater variability (up to 50% higher SD), we aimed to recruit 16 dogs with the goal of obtaining complete data sets from at least 12 dogs in order to maintain a similar power and assuming some attrition related to owner compliance and unexpected co‐morbidities. (http://powerandsamplesize.com/Calculators/Compare‐2‐Means/2‐Sample‐Equality).

Data was assessed for normality using visual inspection of histograms and the Shapiro–Wilk test. Normally distributed data are presented as mean ± SD. Otherwise, data are presented as median (range). All variables were compared between baseline and post treatment with paired tests (paired *t*‐test if normally distributed (i.e., IL‐8, white blood cells, lymphocytes, neutrophils)), or Wilcoxon signed‐rank test if not normally distributed (i.e., cPLI, LPS, IL‐2, IL‐6, TNFα, CK, AST, ALT, triglycerides, cholesterol). For the effect size of serum LPS, serum triglycerides, and serum cholesterol reduction, the percent change from baseline to day 21 was calculated, normality was assessed, and a one sample *t*‐test was performed. Spearman's rho was used to assess correlation between baseline LPS and LPS change from baseline at day 21. Interstitial glucose data was averaged for 6 days during the baseline period (days −6 to −1) and post‐treatment period (days 14–20). Mean IG was compared between baseline and post‐treatment periods using a Wilcoxon test. The difference in changes in amounts (AUC in pixels) of functional classes (i.e., TRL, LDL, HDL) between visits was tested with Friedman test. The difference in amounts (AUC in pixels) of functional classes (i.e., %TRL, %LDL, %HDL), and the nominal subclasses (i.e., %LDL‐1, %LDL‐2, %LDL‐3, %LDL‐4, %LDL‐5, %HDL‐2b, %HDL‐2a, %HDL‐3a, %HDL‐3b, %HDL‐3c) between visits was compared using paired *t*‐tests or Wilcoxon signed rank tests as appropriate. Repeated measure correlations between triglyceride, cholesterol, %TRL, %LDL, or %HDL were examined using r package “rmcorr” [22].

## 3 Results

Sixteen dogs met the inclusion criteria and were enrolled in the study, with a median age of 9.7 years (5.0–12.8), a median body weight of 7.7 kg (5.1–40.0 kg) and a median BCS of 6 out of 9 (5–8 out of 9). There were 8 neutered males and 8 spayed females. The breed distribution consisted of 2 each of miniature poodles, shih tzus, and Yorkshire terriers, and 1 each of Labrador retriever, chihuahua, pug, cocker spaniel, labradoodle, Pomeranian, rat terrier, Australian shepherd, cockapoo, and Hamilton hound‐pit bull mix.

The median time since DM diagnosis at the time of study enrollment was 13 months (range 3–48 months), and at the time of the study, 4 dogs received Novolin N, 4 received Vetsulin, 3 received Toujeo, and 1 each received Levemir, Degludec, Novolin 70/30, a combination of Lantus and Humalog, and a combination of Toujeo and Novolin 70/30. Insulin doses varied at enrollment and are reported in Table S1. Of 16 dogs with data available, based on clinician assessment of FGMS data and clinical assessment, 3 dogs had their insulin dose decreased between the baseline and day 21 visits. Clinical hypoglycemia was not identified in any dog during the entire duration of the study, nor were clinical signs of uncontrolled diabetes reported. There was no difference in 6‐day average interstitial glucose between the baseline period (299 [105–378] mg/dL) and the post‐treatment period (306 [153–357] mg/dL, *p* = 0.1).

The median dose of Tricor was 9.2 mg/kg (7.1–11.0 mg/kg) once daily. All dogs received the Tricor throughout the study period without any major adverse effects. Two dogs vomited (1 or 2 episodes) during the study, one had large bowel diarrhea at the end of the study (but had a history of diarrhea events in the past), and one dog developed Horner's syndrome that was presumably idiopathic. There were no differences between baseline and day 21 for CK, AST, or ALT activities (*p* > 0.05; data not shown). There were also no differences between baseline and day 21 for white blood cell, neutrophil, or lymphocyte counts (*p* > 0.05; data not shown). One dog had a mild neutropenia on day 21 that was not present at baseline; the neutrophil count was within the reference interval (RI) 5 weeks after study completion and cessation of fenofibrate.

**FIGURE 1:**
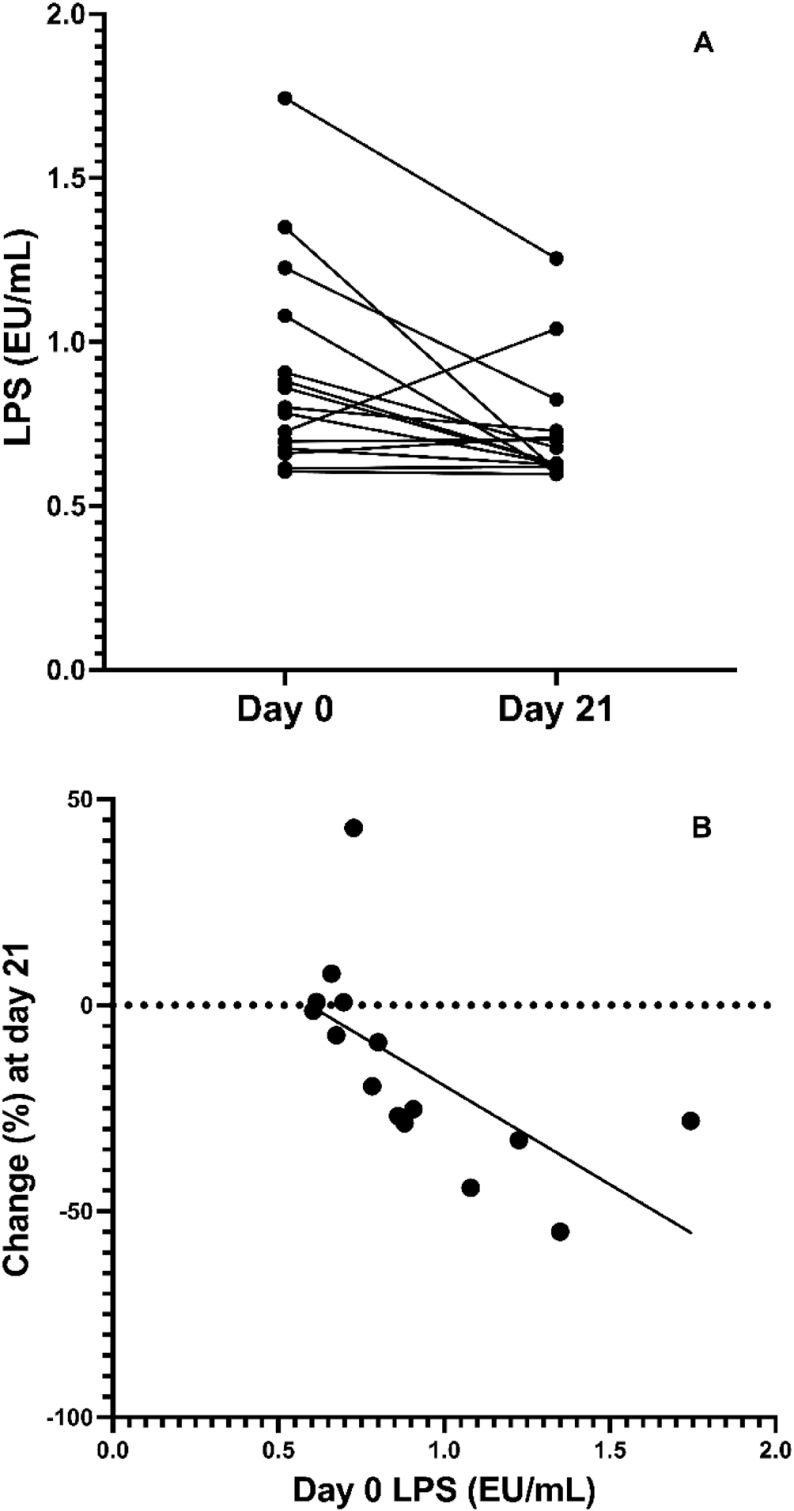
(A) Serum lipopolysaccharide (LPS) for each individual dog at day 0 and after 21 days of fenofibrate treatment (*n* = 15; 1 dog had inadequate serum volume for analysis). (B) Percent change in serum LPS for each individual dog at day 21 of fenofibrate compared to baseline. (*n* = 15; 1 dog had inadequate serum volume for analysis).

There was no difference in the median cPLI at baseline (54 μg/L [29–1390]) compared with day 21 (52 μg/L [29–1015], *p* = 0.72). Additionally, there was no difference between baseline and day 21 values for IL‐2 (*p* = 0.76), IL‐6 (*p* = 0.38), IL‐8 (*p* = 0.18), or TNFα (*p* = 0.42). Serum for 15 dogs was analyzed for LPS due to insufficient sample for one dog. The median LPS at day 21 (0.63 EU/mL [0.60–1.26]) was significantly decreased from baseline (0.80 EU/mL [0.61–1.74], *p* = 0.0172, Figure 1) by 15% ± 24% (95% CI 2–28%, *p* = 0.03). In 11 of the 15 dogs, LPS decreased. In 8 of them, it decreased by more than 20%. In the 4 dogs in which LPS increased, only 1 had increased by more than 20%. The change in LPS was strongly correlated to the baseline values (*r* = 0.85, 95% CI 0.6–0.95, *p* < 0.0001), that is, the higher LPS was at baseline, the more it tended to decrease after fenofibrate treatment.

Serum triglyceride concentrations at day 21 (median 105 mg/dL [43–491]) were significantly decreased from baseline values (median 186 mg/dL [40–1536]) by 36 ± 39% (95% CI 15–56%, *p* = 0.002). Similarly, serum cholesterol concentrations at day 21 (331 ± 99 mg/dL) were significantly decreased from baseline values (421 ± 134 mg/dL) by 20 ± 14% (95% CI 12–28%, *p* < 0.0001). Serum triglyceride concentrations were increased above the upper limit of the RI (ULRI) in only 5 of the 16 dogs at baseline. In 3 of these 5, serum triglyceride concentrations normalized at day 21. One dog that had normal triglyceride concentrations on day 0 had an increase in triglycerides above the ULRI on day 21. In total, 3 of the 16 (19%) dogs had TG above ULRI on day 21. Serum cholesterol concentrations were increased above the ULRI in 12 of the 16 dogs at baseline. In 6 of these 12 dogs, cholesterol normalized at day 21. In total, 6 of the 16 (38%) dogs had cholesterol above the RI on day 21; 3 of these had also increased serum triglycerides.

**FIGURE 2:**
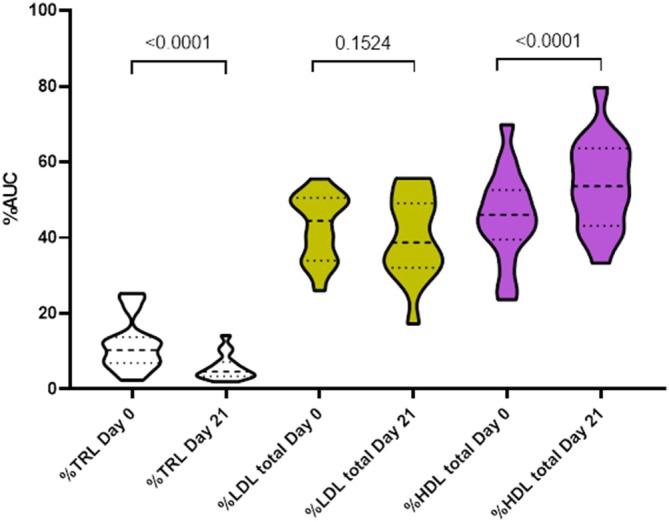
Violin plot of % area under the curve for triglyceride‐rich lipoprotein (TRL), low density lipoprotein (LDL), and high‐density lipoprotein (HDL) in 16 diabetic dogs before (day 0) and after (day 21) treatment with fenofibrate. The thick and thin dashed lines represent the median and quartiles, respectively. The unadjusted *p*‐values are presented. Mean total AUC = (TRL + LDL + HDL): Day 0 = 10 249 = (1272 + 4498 + 4480); day 21 = 8914 = (537 + 3719 + 4658).

**FIGURE 3:**
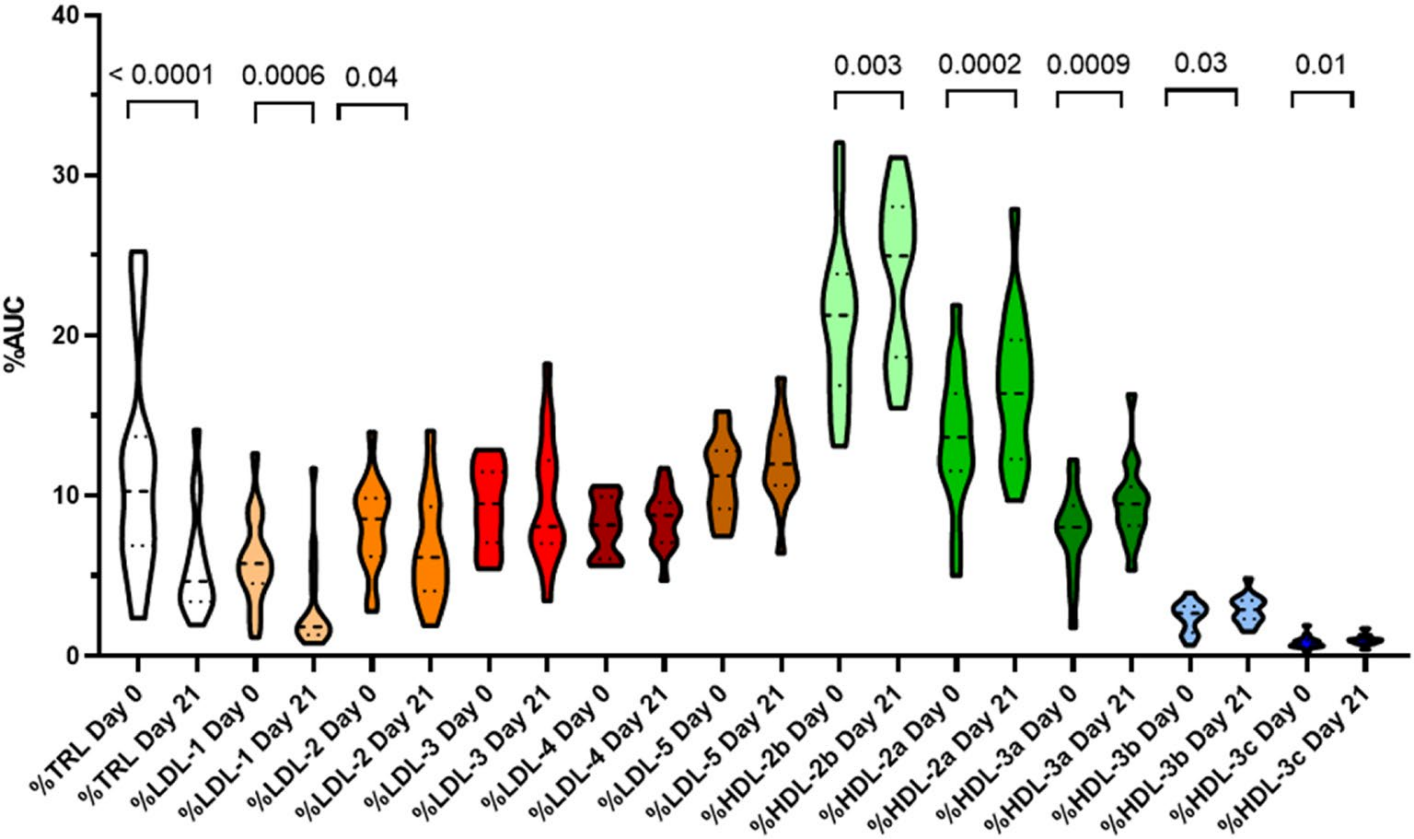
Violin plot of %area under the curve (AUC) for lipid subclasses in 16 diabetic dogs before (day 0) and after (day 21) treatment with fenofibrate. The thick and thin dashed lines represent the median and quartiles, respectively. TRL, Triglyceride‐rich lipoprotein (includes chylomicrons, very‐low‐density lipoproteins, and remnants); LDL, Low‐density lipoprotein; HDL, High‐density lipoprotein. Unadjusted *p*‐values are presented for paired comparisons that are statistically significant (*p* < 0.05). Mean total AUC: Day 0 = 10 249; day 21 = 8914.

The reduction in serum triglyceride concentrations from baseline to day 21 was associated with difference in changes of AUC amounts of lipoprotein functional classes (*p* < 0.0001): selectively reduced median TRL and LDL amounts (AUC) by 53.2% (−82.0% to −5.2%) and 20.6% (−56.3% to 37.0%), respectively while increasing median HDL amounts by 2.6% (−10.3% to 37.0%). The significant changes in serum lipids were associated with changes in the fractional distribution of serum total lipoproteins as mean %TRL decreased by 50.6% (*p* < 0.0001), and mean %LDL by 6% (*p* = 0.1524). Notably, mean %HDL increased by 18.5% (*p* < 0.0001; Figure 2). Additional shifts in lipoprotein subclass distribution occurred in response to decreased TRL amounts. Namely, both %LDL‐1 and %LDL‐2 were reduced (*p* = 0.0006 and 0.04, respectively), following fenofibrate treatment while all %HDL subclasses were significantly increased after treatment (Figure 3). The %TRL was positively correlated with serum cholesterol concentration (Rrm = 0.87, *p* < 0.0001) across all data, rendering TRL the main contributor to the changes in plasma cholesterol concentrations.

## 4 Discussion

The key finding from this study is that dogs with clinically stable, insulin‐treated, spontaneous diabetes mellitus had decreased serum lipids (including total triglycerides, total cholesterol, and %TRL) and LPS after 21 days of fenofibrate therapy, while HDL amounts (AUC) and %HDL both increased. However, there was no change in markers of systemic and pancreatic inflammation (i.e., serum cytokines and cPLI) and no change in mean interstitial glucose in these dogs.

In contrast to a previous dose escalation study [9] that reported 100% normalization of serum triglycerides with fenofibrate treatment at an optimized dose in non‐diabetic dogs, almost 20% of dogs in our study did not have a complete normalization of their serum triglyceride concentrations. This failure rate is similar to another study that used a fixed dose of fenofibrate of 10 mg/kg [23]. In a previous study that showed a reduction of inflammatory cytokines in a canine induced‐diabetes model, fenofibrate was administered by laboratory personnel, not by pet owners, and serum triglycerides decreased in all dogs. It is possible that the failure rate reported here is related to the fixed dose, less‐than‐optimal client compliance, relatively short duration of treatment, or is a feature of spontaneous DM in dogs. Regardless, if normalization of triglyceride concentrations is used as a marker of overall treatment efficacy, it is plausible that the lack of improvement in serum cytokine and cPLI concentrations in our study is related to less‐than‐optimal fenofibrate administration, or to individual variability in response to fenofibrate treatment.

Alterations to the gut microbiota, gut barrier dysfunction, and LPS translocation into the bloodstream are described in diabetic dogs [7, 8]. As circulating LPS is an indicator of intestinal barrier dysfunction [24], our results support the previous study in experimentally induced diabetic dogs that found enhanced markers of intestinal epithelial barrier function after treatment with fenofibrate [7]. In that study, LPS was one of several markers of intestinal barrier function, among a variety of histopathological and immunohistochemical assessments. In our study, intestinal biopsies and direct intestinal permeability tests were avoided to minimize the burden on dogs and their owners. With the absence of corroboration by other intestinal barrier markers, the effect of fenofibrate on LPS should be interpreted with caution, especially considering the variability in LPS responses among individual dogs. On the other hand, the strong correlation between LPS concentration at day 0 and the magnitude of change in LPS gives credence to the suggestion that LPS concentrations represent a biological effect of fenofibrate. Regardless, it is clear that there is a need for additional study in larger numbers of dogs with naturally occurring DM and with additional measures of gut barrier dysfunction.

There was no change in serum cPLI concentrations after fenofibrate treatment in our study, suggesting no effect of fenofibrate on pancreatic inflammation. However, at baseline, only 2 dogs had a Spec cPL > 400 μg/L, consistent with pancreatitis. Future studies might explore the effect of fenofibrate specifically in dogs presented for pancreatitis or hyperlipidemia. As pancreatitis is difficult to definitively diagnose [25], it is possible that we were unable to detect an improvement in pancreatic inflammation with the methods used. In people, hypertriglyceridemia is considered an important cause of acute pancreatitis and accounts for about 10% of all pancreatitis cases [26]. In dogs, a cause and effect relationship has not been established, but the association between hyperlipidemia and pancreatitis has been described [27], and particularly in DM [5]. Considering this association and the evidence from people, it is plausible that hyperlipidemia might sometimes cause pancreatitis in dogs as well. While improved gut‐barrier function was demonstrated after just 3 weeks of fenofibrate treatment, it is possible that pancreatic abnormalities might take longer to respond, explaining the lack of improved cPLI concentrations for the duration of our study. Alternatively, the normal‐to‐moderate levels of hyperlipidemia at baseline in dogs enrolled in our study might have been insufficient to cause pancreatic damage and associated elevated cPLI values; in such a scenario further reduction in serum cPLI values would not be expected. Interestingly though, when compared to healthy dogs, both dogs with overt pancreatitis [27] and the DM dogs in the present study showed elevated TRL amounts and %TRL as well as reduced HDL amounts and %HDL. Similar changes have been reported in human diabetic patients [28], and there are many links between pancreatitis and DM [29]. It might be that the elevated HDL amounts in dogs (normal and diabetic) compared to humans provide protection from overt pancreatitis [30]. Interestingly, TRL appeared to be the primary contributor to untoward hypercholesterolemia in DM dogs. Fenofibrate mediated reductions in TRL associated triglyceride and TRL associated cholesterol in parallel and, in so doing, increased the proportion of plasma cholesterol carried in HDL rather than TRL. Fenofibrate treatment improved glycemic control in type 2 DM and slowed retinal damage in diabetic humans [31, 32]. Although the incidence of DM‐associated ocular complications in dogs is not well‐described, it is common and represents a major source of morbidity [33].

To simplify study design and to avoid using a group of client‐owned dogs treated with placebo, we chose to use dogs as their own controls. To do that, we sought to treat dogs with relatively stable and uncomplicated disease, to allow for a fair comparison between “before” and “after” treatment, isolating “treatment” as the only factor responsible for any potential difference. To achieve this, we excluded cases with concurrent disease, including dogs with signs of chronic, intermittent GI disease and dogs suspected of having pancreatitis (either chronic or acute). By doing so, we likely selected against patients that might benefit more from treatment with fenofibrate. Intestinal inflammation is present in dogs with experimentally induced DM as well as spontaneous, naturally occurring DM [7]. However, in that study, there was no attempt to correlate the degree of intestinal inflammation with clinical outcomes and in fact, at least in the group of dogs with experimentally induced DM, no signs of GI disease were apparent. Although intestinal inflammation as well as systemic markers of inflammation improved after fenofibrate treatment in that study, it is likely that generally, the magnitude of effect of fenofibrate will be smaller in a population of dogs with milder intestinal inflammation. This is supported by our observation that baseline LPS correlated with the magnitude of change in LPS after fenofibrate treatment. It is therefore possible that by selecting cases with no signs of GI disease we decreased the fraction of cases suffering from intestinal inflammation, or selected only cases with milder intestinal inflammation, hence decreasing the chance of observing an effect of treatment. In future studies, the effect of fenofibrate should be tested against placebo in a cohort of dogs with signs of chronic gastrointestinal disease or with evidence of pancreatic inflammation.

Another potential limitation of our study is the lack of control for the potential contribution of periodontal disease to systemic inflammation. Periodontal disease contributes to insulin resistance in dogs with DM, likely by causing a degree of systemic inflammation [34]. As such, it is possible that the lack of apparent response to fenofibrate in terms of serum cytokine concentrations was the result of masking such an effect by inflammation caused by periodontal disease.

Similar to previous studies on the use of fenofibrate in dogs, no clinically relevant adverse effects were reported. Considering the safety of fenofibrate and the benefit it confers on gut barrier function and improved lipid profiles, we speculate that long‐term treatment of diabetic dogs would result in a decreased risk for comorbidities, including gastrointestinal disease and pancreatitis. Considering the burden of diabetes in general on dogs and their caretakers, and in particular the added burden caused by comorbidities of the GI tract (including interfering with insulin therapy, added cost, etc.) any reduction of that burden might have important long‐term benefits to the survival and quality of life of the diabetic dog. The role of fenofibrate as a long‐term adjunct treatment for diabetes mellitus in dogs should therefore be further explored.

## Disclosure

Authors declare no off‐label use of antimicrobials.

## Ethics Statement

Approved by the University of Florida Institutional Animal Care and Use Committee (#202011118) and the Veterinary Hospital Research Review Committee. Authors declare human ethics approval was not needed.

## Conflicts of Interest

The authors declare no conflicts of interest.

## Supporting information


**Table S1.** Body weight and insulin regimen (dose, frequency, and type) for 16 dogs at study enrolment.
