# Supplementary material for: Effect of Fenofibrate on Markers of Gut Barrier Function in Dogs With Naturally Occurring Diabetes Mellitus
Source: J Vet Intern Med. 2025 May 19;39(3):e70125. doi: 10.1111/jvim.70125 (PMC12086328; doi:10.1111/jvim.70125)
Supplement: Supplementary file 1 — Table S1. Body weight and insulin regimen (dose, frequency, and type) for 16 dogs at study enrolment. [file JVIM-39-e70125-s001.docx]

**Supplemental Table 1:** Body weight and insulin regimen (dose, frequency, and type) for 16 dogs at study enrolment.

| **Dog’s**  **Body Weight (kg)** | **Insulin Dose and Frequency** | **Insulin type** | **Total insulin dose (U/Kg/Day)** |
| --- | --- | --- | --- |
| 39.7 | 30U BID | Novolin N | 1.5 |
| 40.0 | 8U BID | Levemir | 0.4 |
| 5.3 | 3U BID | Vetsulin | 1.1 |
| 6.7 | 8U BID | Vetsulin | 2.4 |
| 8.4 | 1.5 U BID  0.75 U BID | Lantus  Humalog | 0.5 |
| 7.9 | 23U once daily | Toujeo | 2.9 |
| 17.9 | 11U BID | Novolin N | 1.2 |
| 14.3 | 28U BID | Toujeo | 3.9 |
| 10.1 | 12U once daily | Toujeo | 1.2 |
| 4.6 | 1.5-2U (AM), 2.5-3U (PM) based on appetite and glucose readings | Novolin N | 1.0 |
| 6.7 | 17U once daily  1U (AM) if eats | Toujeo  Novolin 70/30 | 2.7 |
| 7.4 | 2U AM, 2U mid-day, 3U bedtime | Novolin 70/30 | 0.9 |
| 6.0 | 3U BID | Vetsulin | 1.0 |
| 17.1 | 11 U (AM), 10U (PM) | Novolin N | 1.2 |
| 5.1 | 2U once daily | Degludec | 0.4 |
| 6.6 | 9U BID | Vetsulin | 2.7 |
